# Case of Hypertrophy and Extensive Disease of the Right and Absence of the Left Kidney

**Published:** 1852-05

**Authors:** U. P. Golliday

**Affiliations:** Lacon


ART. II.—*Case of Hypertrophy, and extensive disease of the right, and absence of the left Kidney.*

*Messrs. Editors*—THE following case should have been reported at an earlier date, but various duties caused its delay from time to time. It was peculiar, from the obscurity attending it; and derives interest from the very singular malformation revealed by the autopsy.

Capt. Wm. J. Cline, aged 41—the subject of this case—was visited by me for the first time on the 12th of May, 1850. I found him laboring under the usual symptoms of a mild form of bronchitis. On inquiry, it was ascertained that his health had not been good for some two years—that he had taken a cold two or three weeks previously, but had partially recovered, so as to be able to superintend the removal of his family from Chicago to Henry, in Marshall County. The fatigue and exposure attendant on the journey, had caused a fresh accession of disease.

The bronchial affection seemed to yield readily, but he remained feeble, and soon began to sink gradually under increasing indisposition, which could not have been anticipated from the slight nature of the attack. He finally died, July 2.

As no practical benefit would result from a detailed account, a condensed outline only of general symptoms will be given. Drs. Boal, of Lacon, and Baker, of Henry, were associated with myself attendance. Others were occasionally called in consultation.

The most prominent symptoms were great debility—want of appetite—variable pulse, sometimes full, frequently small and feeble, never strong, corded or wiry—chills, at intervals varying from two or three to eight or ten days, sometimes slight, at others more or less intense, and continuing frequently two or three days, each recurring paroxysm leaving him more debilitated than the previous—urine, passed with difficulty and pain, during the last eight or ten days of life, scanty, purulent, and tinged with blood. He frequently complained of a sensation of weight or stricture in the gastro-umbilical region—never of soreness or pain, unless pressure was made to a greater degree than would seem necessary to detect inflammation in that region.

The following was the result of the autopsy made twenty hours after death—present, Drs. Paddock of Princeton, and Baker of Henry. Countenance, natural; body, emaciated; lungs, sound; heart, normal. In the abdomen, the effects of disease were extensive and well marked. The contiguous surfaces of the peritoneum investing the stomach, transverse arch of the colon, liver, kidney, and spleen, were firmly united by layers and bands of coagulable lymph, in some places firm and evidently of an old date, in others of recent formation and easily separable. Spleen, enlarged and rather firmer than natural. Liver, enlarged; portions of the right lobe slightly indurated; left, soft and easily torn. Kidneys—left, absent; right, enormously enlarged and its pelvis dilated into an immense sac filled with purulent urine. About four inches of the renal extremity of the ureter was dilated to the diameter of an inch, terminating in a cul de sac. The canal proper of the ureter, which would scarcely admit the passage of a small probe, arose from the side of the sac, about an inch from its cystic extremity. Bladder, mucous membrane in some places slightly inflamed. The peritoneal inflammation did not appear to have extended to the mucous membrane of the intestines. The old adhesions of the peritoneum in all probability occurred two years before, at the time of his former illness, which, as I was informed by Mrs. C——, was similar to the disease of which he died.

Peritoneal inflammation may, in general, be easily detected; but in this case it was not suspected during life—probably because the narcotic influence of the urea on the nervous centres, prevented a response by pain to the diseased action going on.

Experience has shown that, when any functional disturbance occurs which has a tendency to impoverish the blood, either by failing to supply its healthy constituents, or to excrete the effete azotized matters taken up in the course of its circulation, local inflammations are exceedingly apt to occur.

In this case, we may rationally suppose the nerves, having been, as it were, overwhelmed by the narcotic influence of the urea, failed to give notice by pain of the local inflammation, and it was left to go on in its course, pouring out lymph and agglutinating contiguous surfaces, as shown by the post mortem. In this way we may account for nearly the whole series of symptoms presented during life. But who would have suspected the existence of but one kidney? We certainly did not; and if it had been suspected, how could death have been prevented, or life prolonged? Here art and skill were powerless. Nothing could be done but endeavor to relieve suffering, by meeting symptoms with what seemed to be appropriate remedies. U. P. GOLLIDAY, M. D.

LACON, March 5, 1852.

